# Pharmacists’ Knowledge, Attitudes, and Practices and Healthcare Stakeholders’ Perspectives on Clinical Pharmacy Services in Referral Hospitals in Mainland Tanzania: A Mixed-Methods Study

**DOI:** 10.7759/cureus.113587

**Published:** 2026-07-29

**Authors:** Ritah F Mutagonda, Manase Kilonzi, Dorkasi Mwakawanga, Alphonce I Marealle, Hamu J Mlyuka, Wigilya P Mikomangwa, Wema A Kibanga, Bertha Mallya, Deogratias Katabalo, Sofia Sanga, Fredrick Kalokola, John Rwegasha, Rose Magambo, John Mmasy, Sungwa Kabissi, Josephine A Balati, Peter Maduki, Appolinary R Kamuhabwa, Omary Minzi

**Affiliations:** 1 School of Pharmacy, Muhimbili University of Health and Allied Sciences, Dar es Salaam, TZA; 2 School of Nursing, Muhimbili University of Health and Allied Sciences, Dar es Salaam, TZA; 3 School of Pharmacy, Catholic University of Health and Allied Sciences, Mwanza, TZA; 4 School of Medicine, Catholic University of Health and Allied Sciences, Mwanza, TZA; 5 Internal Medicine, Muhimbili National Hospital, Dar es Salaam, TZA; 6 Health Policy, Christian Social Services Commission, Dar es Salaam, TZA; 7 Strategic Management, Christian Social Services Commission, Dar es Salaam, TZA

**Keywords:** attitude, clinical pharmacy services, knowledge, pharmacists, practice

## Abstract

Background

Clinical pharmacy services (CPS) support safe, effective, and rational medicine use. In Tanzania, CPS is a core professional responsibility of pharmacists, but evidence on hospital implementation remains limited. This study assessed pharmacists’ knowledge, attitudes, and self-reported practices and explored stakeholder perspectives on CPS implementation in referral hospitals.

Methods

A cross-sectional mixed-methods study was conducted in 17 regions of mainland Tanzania during August-September 2021. The quantitative component included 251 pharmacists. The qualitative component included in-depth interviews of 14 hospital administrators and focus group discussions of 83 pharmacists, medical doctors, and nurses. Quantitative data were analysed using IBM SPSS Statistics for Windows, version 23 (IBM Corp., Armonk, New York, United States), while qualitative data were analysed thematically.

Results

Most pharmacists had adequate knowledge of CPS (96.0%), whereas 48.6% had negative attitudes and 47.8% reported poor practice. Prescription review and dispensing-related activities were reported more frequently than ward-round participation, treatment planning, and clinical documentation. Facility level was associated with practice; pharmacists in regional referral hospitals had a lower prevalence of good practice than those at the national hospital (adjusted prevalence ratio (aPR)=0.43, 95% CI: 0.33-0.55; p<0.001). Qualitative findings confirmed recognition of pharmacists’ clinical value but identified staffing shortages, workload, weak institutional integration, and limited operational guidance as major barriers. Doctors and nurses generally viewed pharmacists’ involvement in direct patient care positively.

Conclusion

Pharmacists demonstrated high awareness and conceptual understanding of CPS, but comprehensive implementation remained limited. Strengthening CPS will require adequate staffing, institutional support, clearly defined clinical roles, practical training and mentorship, operational guidance, and structured interprofessional collaboration.

## Introduction

Healthcare systems are increasingly adopting interprofessional models of care to improve patient outcomes, optimize resource use, and reduce healthcare costs. Within these models, pharmacists are expanding their roles beyond medicine procurement and dispensing to participate directly in patient care through clinical pharmacy services (CPS) [1]. CPS includes medication therapy review, medication reconciliation, therapeutic monitoring, participation in multidisciplinary treatment planning, identification and prevention of medication-related problems, and provision of medicines information. Through these activities, pharmacists support the safe, effective, and rational use of medicines.

Evidence indicates that pharmacists’ participation in direct patient care can reduce medication errors, adverse drug reactions, hospital length of stay, treatment costs, and physician workload [2,3]. Collaborative care involving pharmacists, physicians, and nurses has also been associated with improved medication management and patient satisfaction [2]. Systematic reviews from low- and middle-income countries further suggest that CPS can improve clinical outcomes, medication adherence, health-related quality of life, and health-service utilization while reducing healthcare expenditure [4,5].

Effective CPS implementation depends on pharmacists’ clinical competence, professional attitudes, institutional support, and integration into multidisciplinary teams [6]. Common implementation barriers include inadequate staffing, insufficient clinical training, unclear role expectations, limited infrastructure, and weak organizational support [7-10]. Studies from sub-Saharan Africa suggest that awareness of CPS does not always translate into routine clinical practice. For example, a Nigerian study reported gaps in pharmacists’ knowledge, attitudes, and implementation of pharmaceutical care [11]. These findings highlight the need to examine both individual preparedness and institutional conditions affecting CPS delivery.

Tanzania has introduced educational and regulatory measures to strengthen CPS. Clinical pharmacy content has been incorporated into undergraduate pharmacy training, and postgraduate programmes in clinical pharmacy have been established. In addition, the 2020 amendment to national pharmacy practice regulations formally recognized CPS as a core responsibility of pharmacists [12]. The regulations authorize pharmacists to participate in ward rounds, conduct medication reconciliation, provide therapeutic recommendations, monitor medication safety, and provide medicines information to patients and healthcare professionals.

Despite these developments, evidence on the implementation of CPS in Tanzanian hospitals remains limited. In particular, little is known about pharmacists’ knowledge, attitudes, and routine practices or how other healthcare stakeholders perceive pharmacists’ clinical roles. This mixed-methods study therefore had two objectives: first, to assess pharmacists’ knowledge, attitudes, and self-reported practices regarding CPS; and second, to explore perspectives on CPS implementation among pharmacists, medical doctors, nurses, and hospital administrators in referral hospitals in mainland Tanzania.

## Materials and methods

This was a cross-sectional mixed-methods study conducted in 17 regions of mainland Tanzania, representing five geographical zones. These included the Eastern zone (Dar es Salaam and Coast region), Central zone (Dodoma, Singida, Morogoro), Lake zone (Mwanza, Mara, Geita, and Kagera), Northern zone (Tanga, Kilimanjaro, Manyara, and Arusha), and Southern Highlands zone (Mbeya, Songwe, Iringa, and Njombe). These regions were selected based on the presence of public, faith-based, or private referral hospitals, including regional referral hospitals (RRHs), zonal referral hospitals, specialized hospitals, and the national hospital, as well as the availability of pharmacists. Referral hospitals in Tanzania serve as major centers for specialized healthcare delivery and are expected to provide advanced pharmaceutical care services. The quantitative survey was conducted in 26 hospitals, while qualitative data collection was undertaken in five tertiary hospitals, comprising one national hospital and four zonal referral hospitals, to capture diverse institutional experiences regarding CPS implementation.

The study was approved by the Senate Research and Publications Committee, Muhimbili University of Health and Allied Sciences, Dar es Salaam, Tanzania (reference number: MUHAS-REC-05-2022-1132). Permission to conduct the study was also obtained from the Ministry of Health, regional authorities, and respective hospitals that were involved in the study. All participants provided written informed consent. Privacy and confidentiality were observed during data collection, analysis, and report writing. Unique identification numbers were used to conceal the identities of the study participants

Study participants and sample size

A consecutive sampling approach was used to recruit pharmacists for the quantitative component. All eligible registered and intern pharmacists present at the selected hospitals during the data collection period were approached consecutively and invited to participate. In each hospital, the research team first met the facility leadership, who facilitated introductions to the pharmacy, nursing, and medical departments. After explaining the purpose of the study, departmental leaders assisted in identifying eligible participants.

For the quantitative survey, all eligible registered and intern pharmacists available during the study period in the selected hospitals were invited to participate, resulting in a final sample of 251 pharmacists. For the qualitative component, a purposive sampling strategy was used to recruit participants with relevant professional experience and insight into CPS implementation. The qualitative component included a total of 97 participants, comprising 14 hospital administrators who participated in in-depth interviews (IDIs) and 83 healthcare professionals who participated in focus group discussions (FGDs). The FGD participants included 41 pharmacists, 22 medical doctors, and 20 nurses.

Inclusion criteria for qualitative participants included active involvement in clinical service delivery, leadership responsibilities relevant to pharmaceutical or patient care services, and the ability to provide informed perspectives on CPS implementation. Hospital administrators were purposively selected based on their leadership roles, while healthcare professionals participating in FGDs were selected based on their professional roles and experience in hospital-based patient care. A total of 14 IDIs and 10 FGDs were conducted. Participant recruitment for the qualitative component continued until data saturation was achieved, defined as the point at which no new information or themes emerged from subsequent interviews and discussions.

Data collection

Data were collected between August and September 2021 using quantitative and qualitative methods to obtain a comprehensive understanding of CPS implementation from multiple professional perspectives. All participants were approached face-to-face, informed about the purpose of the study, confidentiality measures, voluntary participation, and their right to withdraw at any time without consequences. Written informed consent was obtained from all participants prior to participation.

English-language questionnaires were administered to pharmacists participating in the survey. FGDs and IDIs were conducted in designated private rooms within hospital premises at times convenient to participants. Five members of the research team (RFM, MK, DLM, HJM, and WPM) conducted qualitative data collection, while another team of trained researchers (WAK, BM, RM, JM, SK, AARK, and OMM) administered the quantitative survey.

Survey

The questionnaire (Appendix A) was adapted from instruments used in previous studies [6,13]. The research team reviewed the original items for relevance to the Tanzanian hospital context, consistency with national pharmacy practice regulations, clarity of terminology, and applicability to referral-hospital practice. Items were modified where necessary to reflect the organization and delivery of CPS in Tanzania. The adapted questionnaire was pretested among 10 pharmacists at the Mwananyamala Regional Referral Hospital, Dar es Salaam, Tanzania, to assess comprehension, wording, sequencing, response options, and administration time. The pretest was intended to improve contextual appropriateness and was not considered a formal psychometric validation of the instrument.

Knowledge and attitude items were scored on a five-point Likert scale from 1 to 5, with higher scores indicating greater knowledge or more favourable attitudes toward CPS. Negatively worded attitude items were reverse-coded before composite scores were calculated. Practice items were scored from 1 for “never” to 5 for “always,” with higher scores indicating more frequent performance of CPS activities. Item scores were summed within each domain, producing possible score ranges of 10-50 for knowledge and 13-65 for both attitude and practice. In the absence of validated cut-off points for this context-specific instrument, the median composite score for each domain was used for categorization. Scores at or above the median were classified as adequate knowledge, positive attitude, or good practice, whereas scores below the median were classified as inadequate knowledge, negative attitude, or poor practice. These categories were specific to the study sample and were not interpreted as validated thresholds of professional competence or service performance.

In-depth interviews

A semi-structured interview guide was developed in English (Appendix B) and translated into Kiswahili for conducting IDIs with hospital administrators. The guide included open-ended questions and probes exploring participants’ perspectives on pharmacists’ involvement in CPS, perceived competencies required for CPS provision, key CPS activities, implementation barriers, and perceived benefits to patients and healthcare institutions. Each interview was conducted at a pre-arranged time convenient to participants. Interviews were conducted by two researchers experienced in qualitative data collection, with one leading the interview while the other took notes and managed audio recording. Each interview lasted approximately 30-60 minutes.

Focus group discussions

An FGD guide was developed in English (Appendix C) and translated into Kiswahili. The guide was informed by the study objectives, literature review, and the research team’s prior experience. FGDs explored participants’ experiences and perceptions regarding CPS implementation in hospitals, including pharmacists’ current roles, interprofessional collaboration, and barriers to service delivery. Including multiple professional groups allowed triangulation of pharmacists’ self-reported experiences with perspectives from collaborating healthcare professionals.

Each FGD was facilitated by two trained researchers, with one serving as moderator and the other responsible for note-taking and audio recording. In each participating hospital, one FGD was conducted with pharmacists only, and one with medical doctors and nurses, resulting in a total of 10 FGDs involving 83 participants. Separating pharmacists from other healthcare professionals allowed participants to express their perspectives freely and minimized professional influence during discussions. FGDs were conducted in private designated rooms within hospital premises. Each discussion included 6-10 participants and lasted approximately 60-120 minutes.

Data analysis

Quantitative data were analysed using the IBM SPSS Statistics for Windows, version 23 (IBM Corp., Armonk, New York, United States). Categorical variables were summarized using frequencies and percentages, while continuous variables were summarized using medians and interquartile ranges (IQRs), as appropriate.

Associations between participant and facility characteristics and good CPS practice were initially examined using unadjusted modified Poisson regression with robust standard errors. Variables with a p-value <0.20 in the unadjusted analysis were included in the multivariable model. Crude and adjusted prevalence ratios (aPRs), with corresponding 95% confidence intervals (CIs), were reported. Statistical significance was set at p<0.05. Cluster-adjusted and multilevel approaches were considered but were not applied because of the limited number of hospitals, unequal cluster sizes, sparse representation of some facility categories, and inclusion of only one national hospital. The regression estimates were therefore interpreted cautiously as exploratory associations.

Qualitative data from in-depth interviews and focus group discussions were audio-recorded, transcribed verbatim, and translated into English where necessary. Six members of the research team independently reviewed the transcripts and field notes to become familiar with the data and contextual information. Data were analysed using a hybrid thematic approach that combined deductive and inductive coding [14].

An initial coding framework was developed from the study objectives, interview guides, and concepts emerging during data familiarization. Coding was conducted independently in pairs, and discrepancies were resolved through discussion and consensus. Related codes were organized into subthemes and overarching themes based on recurring patterns and conceptual relationships. The multidisciplinary research team collaboratively reviewed and refined the final themes, which are presented with illustrative participant quotations. Quantitative and qualitative findings were integrated during interpretation by comparing the principal survey findings with corresponding qualitative themes. The analysis examined areas of convergence, complementarity, and divergence to determine how qualitative findings explained or contextualized the quantitative results.

Rigor and reliability

To enhance the credibility and trustworthiness of the qualitative findings, methodological triangulation was employed through the use of surveys, IDIs, and FGDs, alongside participant triangulation involving pharmacists, medical doctors, nurses, and hospital administrators. This approach enabled a more comprehensive understanding of CPS implementation across different professional perspectives.

The multidisciplinary composition of the research team, including experts in pharmacy, clinical practice, midwifery, sociology, qualitative research, and public relations, strengthened interpretation of findings and minimized single-discipline bias. Team-based coding, iterative discussion of emerging themes, and consensus-based interpretation further enhanced analytical rigor and dependability.

## Results

Quantitative findings

Characteristics of Quantitative Survey Participants

A total of 251 pharmacists participated in the quantitative survey. Their median age was 29 years (IQR: 26-35), and 62.2% were male. Most participants (70.5%) were employed in public hospitals, while almost half (47.8%) worked in RRHs. Most participants (92.8%) held an undergraduate pharmacy degree, and approximately half (51.2%) had less than one year of professional experience as pharmacists. More than half (52.2%) were intern pharmacists at the time of the study (Table 1).

**Table 1:** Characteristics of quantitative survey participants (N=251) Data given as frequency and percentage, except where marked as median (IQR). *The data for Age and Experience as a pharmacist were each missing for one participant; percentages for these variables are based on participants with available data (n=250) IQR: interquartile range

Variable		Frequency (Percentage)
Sex	Male	156 (62.2%)
	Female	95 (37.8%)
Age (years)*	Median (IQR)	29 (26 – 35)
	< 30	132 (52.8%)
	30-40	92 (36.8%)
	> 40	26
Facility level	National hospital	31 (12.4%)
	Zonal referral hospitals	83 (33.1%)
	Specialized hospitals	17 (6.8%)
	Regional referral hospitals	120 (47.8%)
Nature of the facility	Faith-based hospitals	56 (22.3%)
	Public hospitals	177 (70.5%)
	Private hospitals	18 (7.2%)
Level of education	Undergraduate	233 (92.8%)
	Postgraduate	18 (7.2%)
Experience as pharmacist (years)*	Median (IQR)	0.8 (0.7 – 5)
	< 1	128 (51.2%)
	1 – 5	63 (25.2%)
	> 5	59 (23.6%)
Experience as pharmacist at the facility (years)	Median (IQR)	0.8 (0.7 – 3)
	< 1	145 (57.8%)
	1-5	69 (27.5%)
	> 5	37 (14.7%)
Professional rank	Intern pharmacist	131 (52.2%)
	Pharmacist	120 (47.8%)
Employment status	Full-time employment	113 (45.0%)
	Internship	131 (52.2%)
	Others	7 (2.8%)

Pharmacists’ Knowledge of Clinical Pharmacy Services

**Figure 1:**
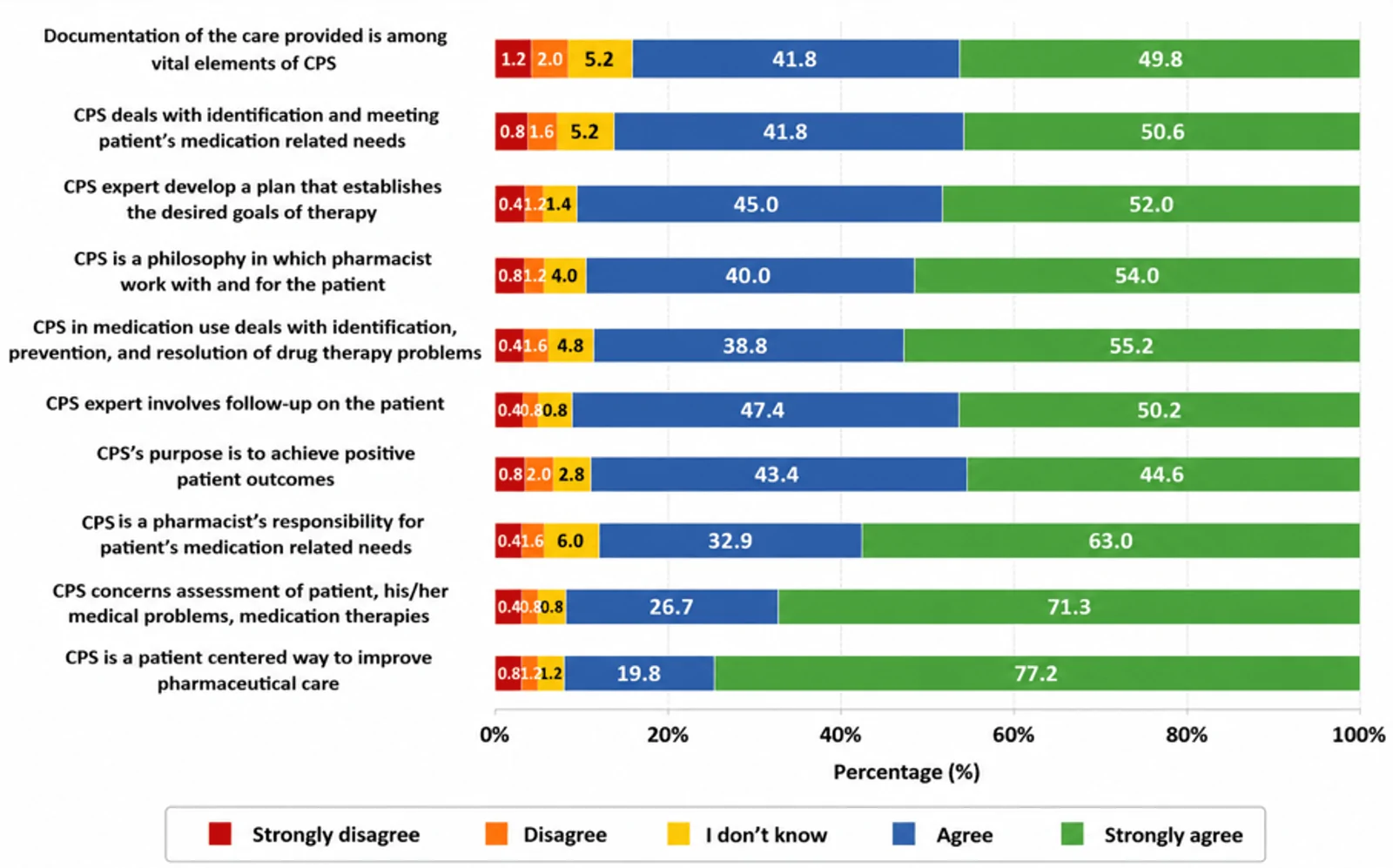
Pharmacists' knowledge regarding CPS CPS: clinical pharmacy services

Figure 1 presents pharmacists’ responses to the knowledge assessment items. Overall, 241 (96.0%) participants demonstrated adequate knowledge of CPS. The majority of participants agreed that CPS is a patient-centered approach aimed at improving pharmaceutical care and therapeutic outcomes. Specifically, 246 (98.0%) agreed that the primary purpose of CPS is to achieve positive patient outcomes, while 243 (96.8%) agreed that CPS is a patient-centered strategy for improving pharmaceutical care. Participants also demonstrated a strong understanding of the clinical scope of CPS. Nearly all participants (n=246, 98.0%) agreed that CPS involves assessment of patients’ medical conditions and medication therapies, while 244 (97.4%) agreed that pharmacists have a responsibility to address patients’ medication-related needs. Similarly, 221 (88.0%) agreed that patient follow-up is an essential component of CPS, and 230 (91.6%) agreed that documentation of care provided is an important element of CPS implementation.

Pharmacists’ Attitudes Toward CPS Provision

**Figure 2:**
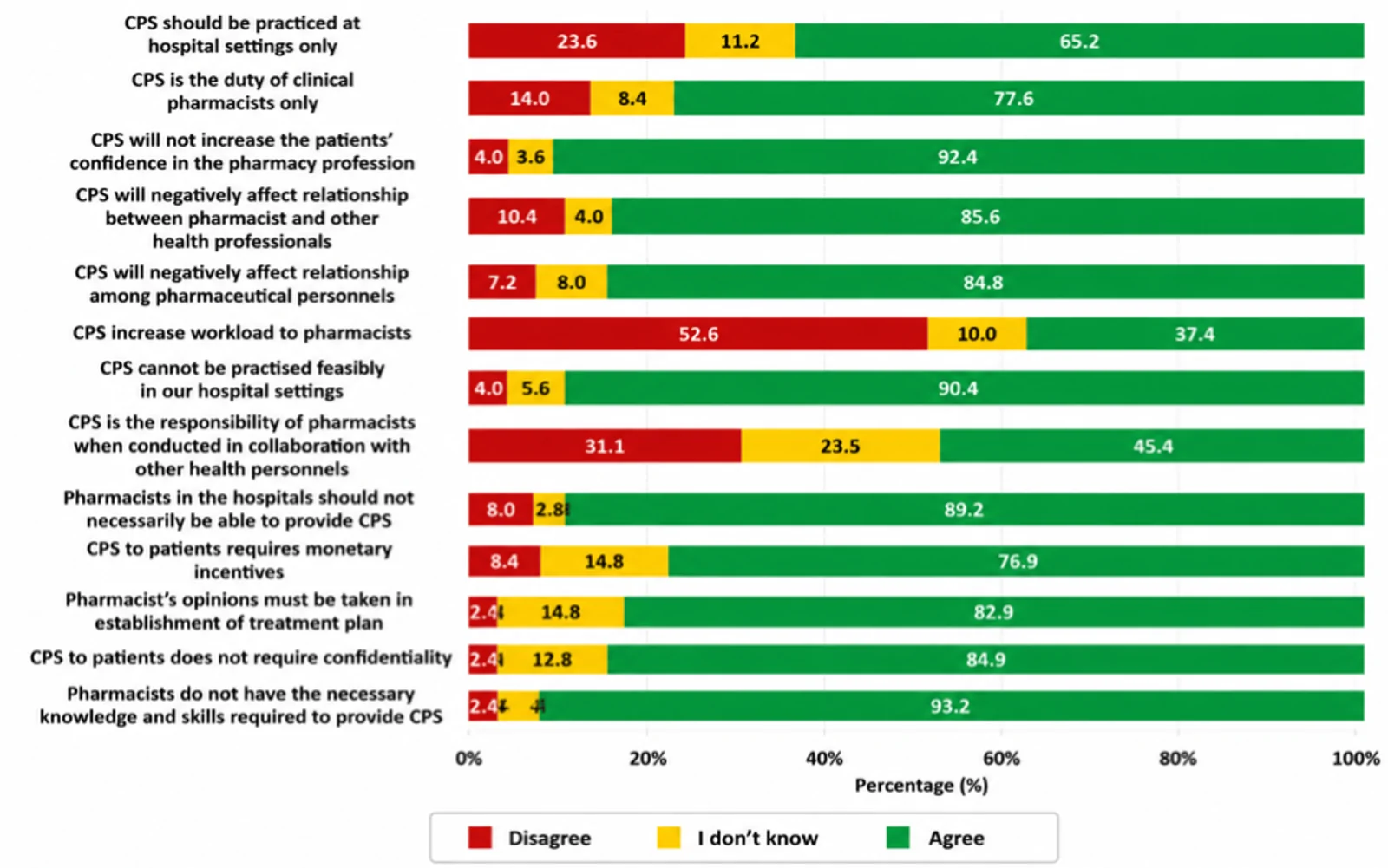
Pharmacists’ attitudes toward clinical pharmacy services

Figure 2 summarizes pharmacists’ attitudes toward CPS implementation. Overall, 122 (48.6%) participants demonstrated a negative attitude toward CPS provision. The most frequently endorsed concerns related to implementation feasibility, workload, professional preparedness, and workplace relationships. Approximately 193 (76.8%) participants agreed that pharmacists may lack sufficient knowledge and skills to effectively implement CPS, while 163 (64.8%) perceived CPS implementation as likely to increase pharmacists’ workload. Concerns regarding institutional feasibility were also evident, with 181 (72.0%) indicating that CPS may not be practically feasible in their current hospital settings. Additionally, many participants expressed concerns about the potential impact of CPS implementation on workplace dynamics, with 235 (93.7%) perceiving possible negative effects on relationships among pharmaceutical personnel and 215 (85.6%) anticipating strain in interprofessional collaboration with other healthcare professionals. Furthermore, 109 (43.4%) indicated that implementation of CPS may require additional financial incentives beyond routine salary support.

Self-Reported Clinical Pharmacy Practice

**Figure 3:**
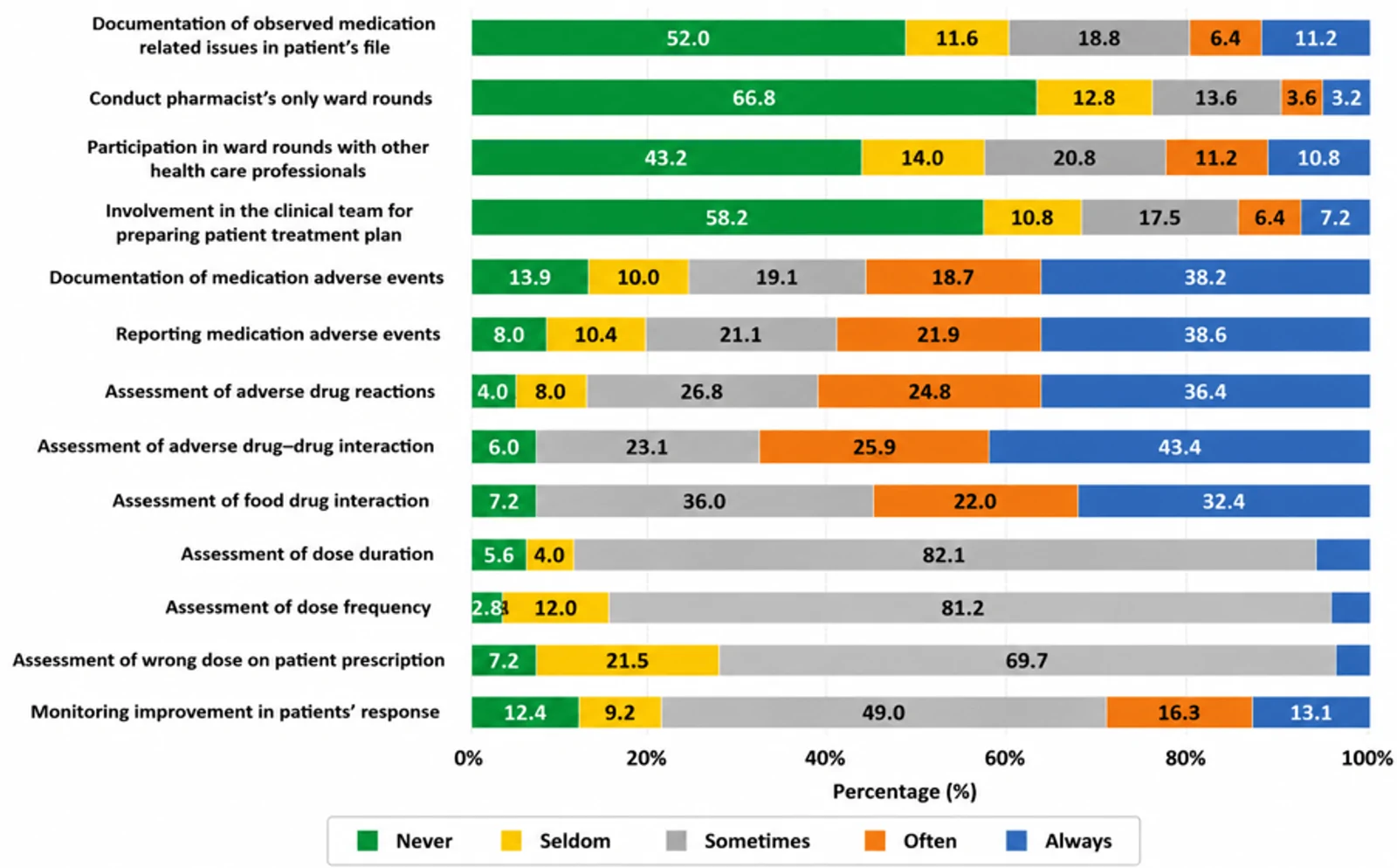
Self-reported clinical pharmacy service practices among pharmacists

Figure 3 presents pharmacists’ self-reported CPS practice. Overall, 120 (47.8%) participants were categorized as having poor CPS practice. Participation in multidisciplinary direct patient care activities was relatively limited. Only 51 (20.4%) reported participating in pharmacist-only ward rounds, while 107 (42.8%) reported involvement in multidisciplinary ward rounds with other healthcare professionals. More than half, 146 (58.2%), reported never participating in the development of patient treatment plans within multidisciplinary clinical teams. Documentation-related practices were similarly limited. Only 91 (36.4%) reported routinely documenting medication-related issues in patients’ clinical records. In contrast, pharmacists reported strong engagement in prescription-focused medication review activities, particularly during dispensing. The most commonly reported activities included assessment of dosing frequency by 234 (93.2%), treatment duration by 232 (92.5%), and identification of incorrect doses in prescriptions by 229 (91.2%) participants.

Factors Associated With Good Practice in the Provision of CPS

Table 2 presents the factors associated with pharmacists’ CPS practice. In the unadjusted analysis, education level, professional experience, and facility level were associated with good CPS practice. In the multivariable analysis, facility level remained associated with CPS practice. Compared with pharmacists working at the national hospital, those working in lower-level referral hospitals had significantly lower prevalence of good CPS practice. Specifically, pharmacists working in zonal referral hospitals had a 38% lower prevalence of good CPS practice (aPR = 0.62, 95% CI: 0.44-0.87; p = 0.007), those in RRHs had a 57% lower prevalence (aPR = 0.43, 95% CI: 0.33-0.55; p < 0.001), and those in specialized hospitals had a 54% lower prevalence (aPR = 0.46, 95% CI: 0.26-0.81; p = 0.007), compared with pharmacists at the national hospital.

**Table 2:** Factors associated with good clinical pharmacy service practice among pharmacists (N=251) *The data for Age and Experience as a pharmacist were each missing for one participant; percentages for these variables are based on participants with available data (n=250) CPS: clinical pharmacy services; RRH: regional referral hospital

Variable	Frequency (Percentage)	cPR (95%CI)	P-value	aPR (95%CI)	P-value
Age group (years)*					
< 30	64/132 (48.5%)	0.79 (0.55-1.12)	0.183	0.99 (0.65-1.51)	0.968
30-40	51/92 (55.4%)	0.91 (0.64-1.30)	0.605	0.94 (0.67-1.32)	0.712
> 40	16/26 (61.5)	Reference	-	Reference	-
Education level					
Undergraduate	117/233 (50.2%)	0.65 (0.49-0.86)	0.002	0.80 (0.56-1.15)	0.226
Postgraduate	14/18 (77.8%)	Reference	-	Reference	-
Experience as pharmacist (years)*					
< 1	58/128 (45.3)	0.67 (0.52-0.88)	0.003	0.75 (0.51-1.10)	0.139
1-5	34/63 (54)	0.80 (0.60-1.07)	0.138	0.87 (0.63-1.22)	0.423
> 5 years	39/59 (66.1)	Reference	-	Reference	-
Facility level					
Zonal	47/83 (56.6%)	0.61 (0.49-0.75)	<0.001	0.62 (0.44-0.87)	0.007
RRH	48/120 (40.0%)	0.43 (0.34-0.55)	<0.001	0.43 (0.33-0.55)	<0.001
Specialized	8/17 (47.1%)	0.50 (0.30-0.84)	0.009	0.46 (0.26-0.81)	0.007
National	28/31 (90.3%)	Reference	-	Reference	-
Ownership of facility					
Faith based	29/56 (51.8%)	0.93 (0.57-1.51)	0.776	-	-
Public	92/177 (52.0%)	0.95 (0.61-1.46)	0.804	-	-
Private	10/18 (55.6%)	Reference	-	-	-
Gender					
Male	86/156 (55.1%)	1.14 (0.88-1.47)	0.313	-	-
Female	45/95 (47.4%)	Reference	-	-	-
CPS knowledge level					
Inadequate	6/10 (60.0)	1.06 (0.83–1.37)	0.630	-	-
Adequate	125/241 (51.9)	Reference	-	-	-
CPS attitude level					
Negative	61/122 (50.0)	0.92 (0.73–1.17)	0.501	-	-
Positive	70/129 (54.3)	Reference	-	-	-

Qualitative findings

Characteristics of Participants in Qualitative Component

A total of 97 participants were included in the qualitative component. Of these, 14 were hospital administrators, including four Executive Directors, five Directors of Medical Services, and five Heads of Pharmacy Departments. The remaining 83 healthcare professionals participating in focus group discussions comprised 41 pharmacists, 22 medical doctors, and 20 nurses. Slightly more than half (54.6%) were male, with participant ages ranging from 25 to 68 years.

Among hospital administrators, educational qualifications ranged from bachelor’s degree to PhD level, and 85.7% had more than three years of leadership experience. Among FGD participants, 61 held bachelor’s degrees, five were medical specialists, eight were clinical pharmacists, and 47 had more than five years of professional experience.

Thematic analysis

Three themes were identified: recognition of pharmacists’ clinical roles and the value of CPS; barriers to pharmacists’ integration into direct patient care; and pharmacists’ existing contributions to medication-related clinical support services. 

Theme 1: Recognition of Pharmacists’ Clinical Roles and the Perceived Value of CPS

Qualifications required for CPS provision: Participants consistently described CPS providers as pharmacists possessing adequate clinical knowledge and competencies in pharmacology, disease pathophysiology, interpretation of laboratory and imaging findings, and medication management. Beyond technical expertise, participants emphasized the importance of strong communication skills, professionalism, ethical conduct, confidence, commitment, and the ability to collaborate effectively within multidisciplinary healthcare teams.

“It is a must to have a pharmacy degree with training in pharmacology and experience in that area… but it is better to have good communication skills because this person must work with other health care workers such as doctors and nurses.” (FGD1AP2)

Roles and activities provided under CPS: Participants demonstrated a clear understanding of expected CPS roles, including participation in treatment planning, medication selection and optimization, dose adjustment, identification of drug-drug, drug-food, and drug-disease interactions, therapeutic monitoring, adverse drug reactions (ADR) reporting, patient counseling, and documentation of medication-related interventions. Participants also emphasized pharmacists’ advisory role in supporting both patients and other healthcare professionals in medication-related decision-making.

“Pharmacists should provide advice… to both the patient and the management team, including advising doctors and nurses when making decisions about medication.” (IDIDS3)

Perceived benefits of CPS: Participants perceived CPS as beneficial at multiple levels, including healthcare institutions, healthcare professionals, and patients. At the institutional level, CPS was viewed as a strategy for improving medication use efficiency, reducing operational costs associated with inappropriate medicine use, enhancing quality of care, strengthening public trust in healthcare services, and potentially creating opportunities for research and institutional growth.

“Firstly, I think when there is collaboration among healthcare workers, the hospital gains trust from the people in healthcare provision. Trust is a huge factor built from the bottom, so it adds so much value to the hospital, making it a source of good and trusted service.” (FGD5BP1)

Healthcare professionals perceived pharmacist involvement in direct patient care as beneficial in reducing workload, strengthening interprofessional collaboration, improving medication-related knowledge among team members, and promoting shared learning and professional development. Participants also noted that greater pharmacist involvement in CPS could enhance professional recognition of pharmacists within clinical care settings.

“One of the benefits of pharmacists’ involvement in CPS is education because, at work, you continuously learn every day and from other people’s experiences as well. Working together between pharmacists, doctors, and nurses creates awareness of CPS and opportunities for healthcare workers to learn from each other in patient care, including the use of medicines.” (IDIMS4)

Participants further reported that pharmacist-led CPS could improve rational medicine use, enhance medication adherence, reduce adverse drug reactions, minimize polypharmacy and medication toxicity, lower treatment costs, shorten hospital stays, and ultimately improve patient outcomes and satisfaction with care.

“Involvement of pharmacists in the provision of CPS results in better patient outcomes, as well as minimizing ADRs that may occur through the use of prescribed medicines in the hospital.” (FGD5AP6)

Although participants generally recognized the potential value of CPS, they described several barriers that limited the translation of these expected roles into routine practice.

Theme 2: Barriers Limiting Pharmacists’ Integration into Direct Patient Care

Limited participation in ward rounds: Participants reported limited and inconsistent pharmacist participation in multidisciplinary ward rounds across most hospitals. While ward-round participation was recognized as an essential component of CPS, pharmacists were described as rarely participating in routine clinical rounds. Where pharmacist involvement existed, it was often restricted to specific departments, particularly internal medicine, and occurred inconsistently. Where ward-round participation occurred, it was often limited to selected departments or depended on local institutional arrangements.

“In the department of internal medicine, there is a pharmacist who does ward rounds once per week to advise on medication provision, changing the medications and things like that.” (FGD2BP2)

In some facilities, participants reported that CPS implementation had been discussed or planned but had not yet been formally operationalized due to unresolved institutional barriers.

“In part, ward rounds have been planned in our hospital, but we have not officially started implementation. There are issues that need to be addressed for the provision of CPS to start.” (FGD5AP3)

Despite these limitations, participants described several medication-related activities that pharmacists were already providing within hospitals.

Theme 3: Existing Pharmacist Contributions in Medication-Related Clinical Support Services

Prescription review and patient counselling: Participants reported that pharmacists were involved in prescription review, identification of medication errors, dose verification, provision of medicines information, and patient counselling. In some hospitals, pharmacists also contributed to antimicrobial stewardship, hospital formulary development, and chemotherapy services. Participants also described pharmacist involvement in selected specialized clinical activities, including antimicrobial stewardship discussions, preparation of hospital formularies, and chemotherapy preparation and administration in collaboration with other healthcare professionals.

“In some cases, pharmacists are involved in advising other HCWs for rational use of antibiotics … I work with a pharmacist who comes to the department of internal medicine for discussion on the use of antibiotics, availability of medicines in the hospital, and antimicrobial resistance.” (FGD2BP2)

“Pharmacists are involved in mixing chemotherapy and are also involved in administering chemotherapy to patients in collaboration with nurses.” (FGD2BP3)

Pharmacovigilance activities: Participants identified pharmacists as important contributors to hospital pharmacovigilance. Reported activities included detecting suspected adverse drug reactions, completing reporting forms, and submitting reports to hospital units or regulatory authorities. Some participants noted that communication and feedback on reported events were not always timely.

“We also follow up on ADRs and fill the forms, or sometimes we electronically send ADR reports to the regulatory authorities.” (FGD5AP7)

However, some participants noted gaps in communication and feedback mechanisms, reporting that ADR findings were not always promptly shared with other healthcare professionals for immediate clinical intervention or collective learning.

## Discussion

CPS is increasingly recognized as a core component of patient-centered healthcare because medicines are central to modern treatment but can also cause preventable harm when medication-use processes are fragmented or inadequately monitored [15,16]. Pharmacists contribute to medication optimization through medication review, therapeutic monitoring, medicines information, pharmacovigilance, medication reconciliation, and identification of medication-related problems [2,17].

In this mixed-methods study, pharmacists demonstrated high awareness and conceptual understanding of CPS, but comprehensive CPS activities were not routinely implemented. Almost half of the surveyed pharmacists had negative attitudes or reported poor practice. Quantitative and qualitative findings converged in showing that CPS delivery was concentrated in prescription review, dispensing-related medication checks, patient counselling, and pharmacovigilance, whereas participation in ward rounds, treatment planning, and clinical documentation was less frequent. The qualitative findings complemented the survey results by identifying organizational and operational factors that may explain the gap between knowledge and routine practice. The two components also revealed divergence in interprofessional perceptions: pharmacists anticipated possible professional resistance, whereas medical doctors and nurses generally viewed pharmacists’ participation in direct patient care positively.

The high proportion of pharmacists classified as having adequate knowledge may reflect changes in pharmacy education and regulation in Tanzania. Clinical pharmacy has increasingly been incorporated into undergraduate and postgraduate training, while national regulations formally recognize CPS as a professional responsibility [7,18]. Similar findings have been reported in settings where pharmacy education reforms have increased exposure to patient-centred care [6,19]. However, the knowledge items mainly assessed awareness and conceptual understanding of CPS rather than advanced clinical competence. Furthermore, the cross-sectional design does not allow the observed knowledge levels to be directly attributed to educational or regulatory reforms.

Despite high knowledge, almost half of the pharmacists were classified as having negative attitudes toward CPS implementation. Their concerns related mainly to preparedness, workload, feasibility, financial expectations, and professional relationships. Comparable concerns have been reported in other low- and middle-income settings, where pharmacists may support the principles of pharmaceutical care but remain uncertain about the feasibility of implementation within existing health-system conditions [6,13,20,21]. Integration of the quantitative and qualitative findings suggests that these attitudes may reflect uncertainty about implementation readiness rather than opposition to CPS, as qualitative participants generally recognized the value of pharmacists’ clinical roles.

An important divergence emerged between pharmacists’ anticipated concerns and the experiences reported by other professional groups. Many pharmacists expected CPS implementation to strain relationships with other healthcare professionals. In contrast, medical doctors and nurses participating in the qualitative component generally regarded pharmacists’ clinical involvement as beneficial for medication-related decision-making, workload sharing, and patient care. Similar findings have been reported in settings where pharmacists make visible and clinically relevant contributions within multidisciplinary teams [21,22]. This divergence suggests that anticipated resistance may partly reflect limited exposure to collaborative practice rather than established opposition from other professional groups.

Self-reported CPS practice was concentrated mainly in prescription review, dose verification, patient counselling, and pharmacovigilance. Participation in multidisciplinary ward rounds, treatment planning, and structured documentation was less common. Similar patterns have been reported in other African hospital settings, where pharmacists remain more involved in medicine-focused support functions than in fully integrated ward-based care [8,20,21]. These findings indicate partial adoption of CPS rather than its comprehensive integration into routine clinical care.

Facility level was associated with CPS practice. Pharmacists working in zonal, regional referral, and specialized hospitals had a lower prevalence of good practice than those working at the national hospital. The qualitative findings complemented this result by identifying differences in staffing, infrastructure, leadership support, clinical practice arrangements, and the formal integration of pharmacists into multidisciplinary teams. Previous studies in Tanzania and other resource-constrained settings have similarly identified organizational readiness as an important determinant of CPS implementation [7,21,22]. However, because clustering of pharmacists within hospitals was not explicitly accounted for, the precision of these facility-level estimates may be overstated. The observed associations should therefore be interpreted cautiously and may partly reflect unmeasured characteristics of individual hospitals.

The findings can be interpreted using the Capability-Opportunity-Motivation-Behaviour framework [23]. Pharmacists demonstrated baseline capability through their awareness of CPS concepts and responsibilities. However, limited institutional opportunities appeared to constrain practice, while concerns about feasibility may have weakened professional motivation. The framework therefore helps explain why knowledge did not consistently translate into routine CPS delivery: implementation requires individual capability, supportive organizational opportunities, and sufficient motivation.

This study has several strengths, including its mixed-methods design, broad geographic coverage, inclusion of pharmacists from different referral-hospital levels, and participation of pharmacists, medical doctors, nurses, and hospital administrators in the qualitative component. These features enabled triangulation of findings and provided contextual insight into CPS implementation. Several limitations should, however, be considered. Practice was self-reported and may have been affected by recall and social desirability bias. The inclusion of intern pharmacists, who may have had limited independent clinical responsibilities, may also have influenced the reported level of practice. Qualitative data were collected only from tertiary hospitals, which may limit transferability to lower-level referral facilities. The regression analysis did not explicitly account for clustering of pharmacists within hospitals, potentially underestimating standard errors and overstating the precision of facility-level associations. In addition, some facility categories included few hospitals, and the national-hospital reference category was represented by a single institution. The facility-level findings should therefore be interpreted as exploratory and may partly reflect unmeasured hospital-specific characteristics. The questionnaire was adapted and contextually pretested but was not subjected to comprehensive psychometric validation in Tanzania. Moreover, the median-based knowledge, attitude, and practice categories were sample-specific and should not be interpreted as established thresholds of competence or performance. Finally, the cross-sectional design precludes causal inference regarding factors associated with CPS practice.

## Conclusions

Among pharmacists surveyed in referral hospitals in mainland Tanzania, awareness and conceptual understanding of CPS were high, but routine implementation of comprehensive CPS activities remained limited. Qualitative findings from pharmacists, medical doctors, nurses, and hospital administrators indicated that inadequate staffing, high workload, weak integration of pharmacists into clinical teams, and the absence of clear operational guidance constrained implementation. Pharmacists’ concerns about interprofessional collaboration contrasted with the generally positive perspectives of medical doctors and nurses regarding pharmacists’ participation in direct patient care.

These findings suggest that knowledge of CPS alone is insufficient to support routine implementation without adequate workforce capacity, institutional commitment, clearly defined clinical roles, practical training and mentorship, and structured interprofessional collaboration. Context-specific implementation frameworks and standard operating procedures may support more effective integration of CPS into hospital practice. Given the cross-sectional design and reliance on self-reported practice measures, the findings should be interpreted as reported implementation patterns and associated barriers rather than evidence of causal effects or improved patient outcomes.

## Figures and Tables

**Figure 4:**
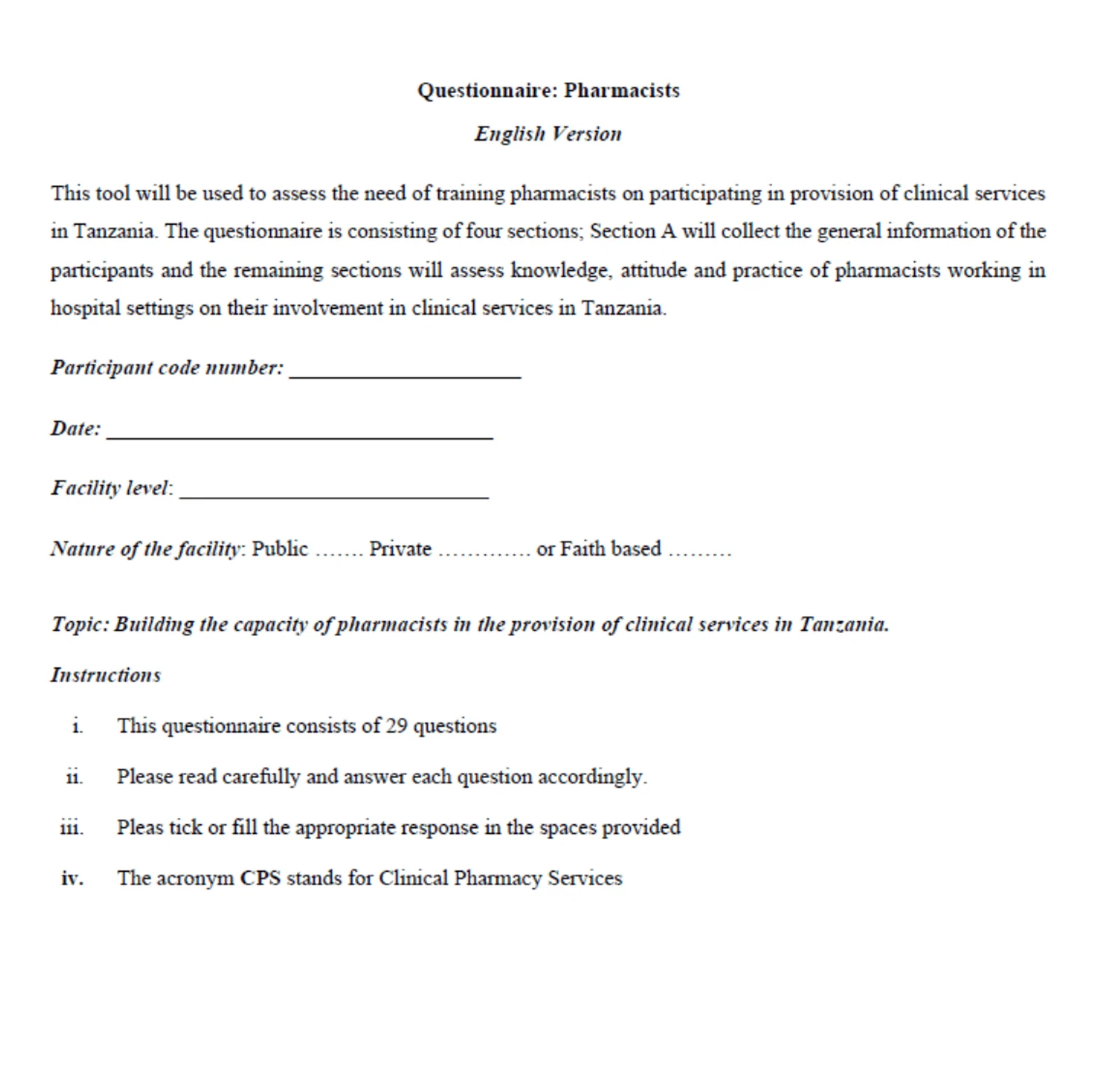
Questionnaire for pharmacists (page 1)

**Figure 5:**
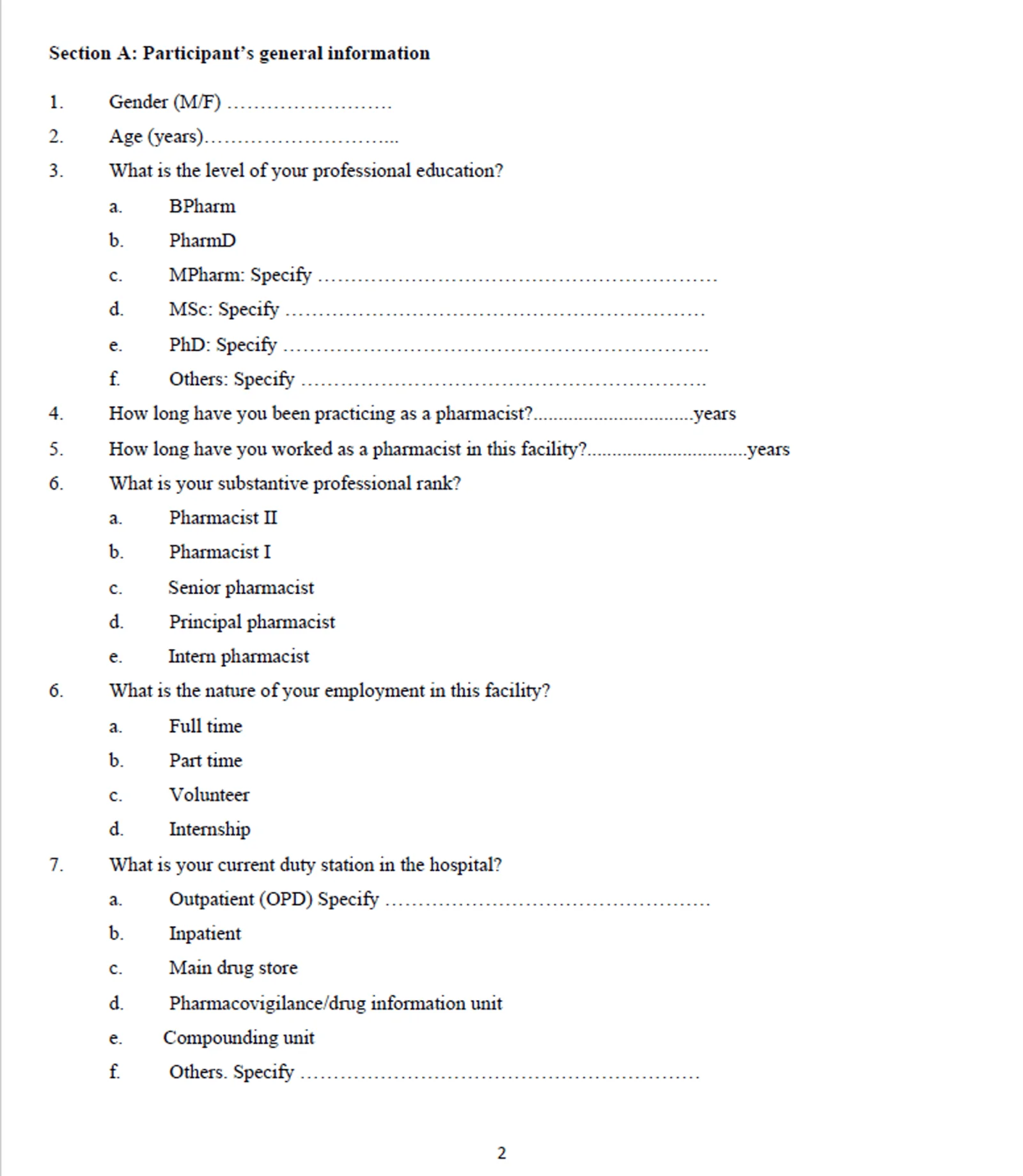
Questionnaire for pharmacists (page 2)

**Figure 6:**
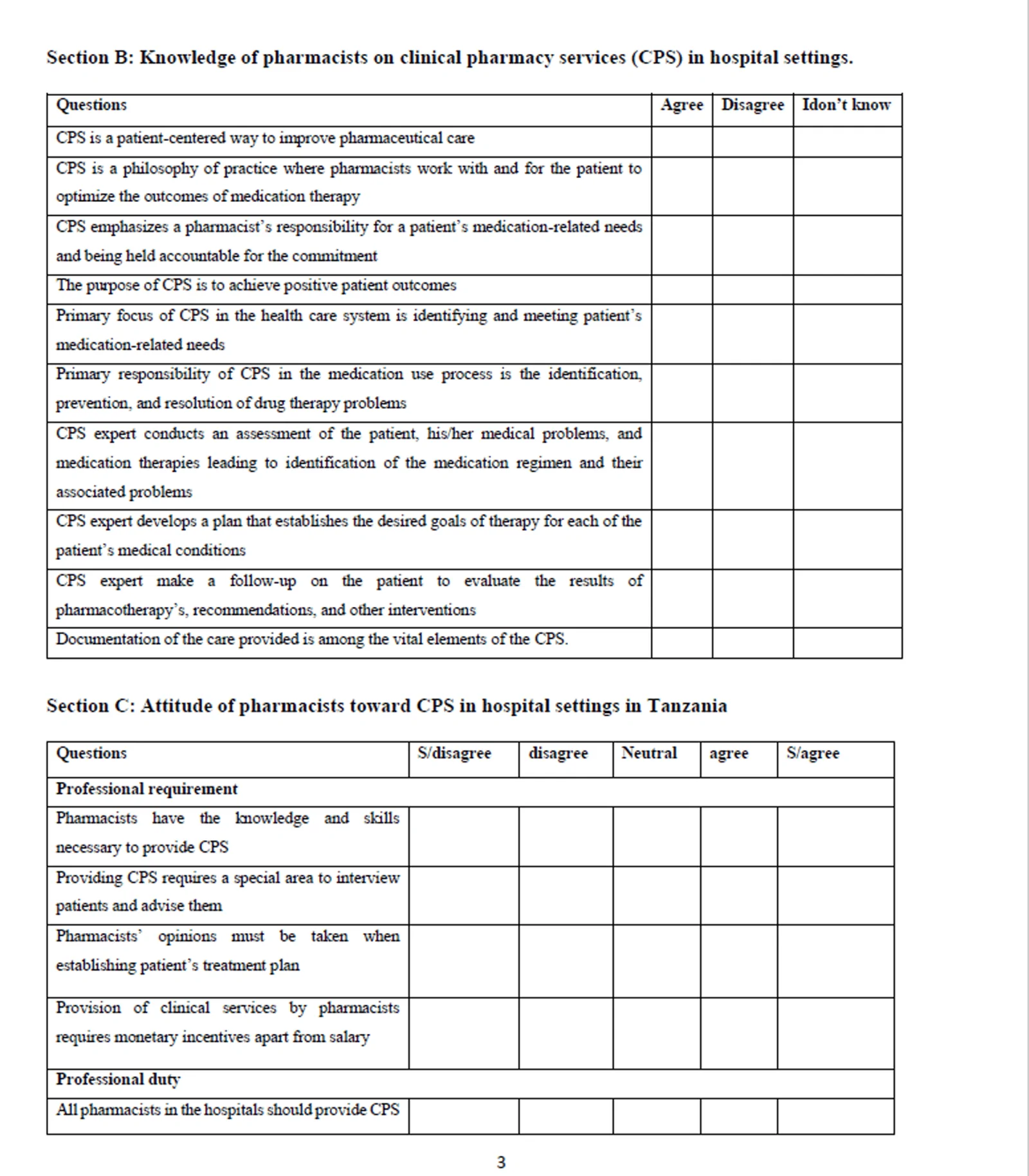
Questionnaire for pharmacists (page 3)

**Figure 7:**
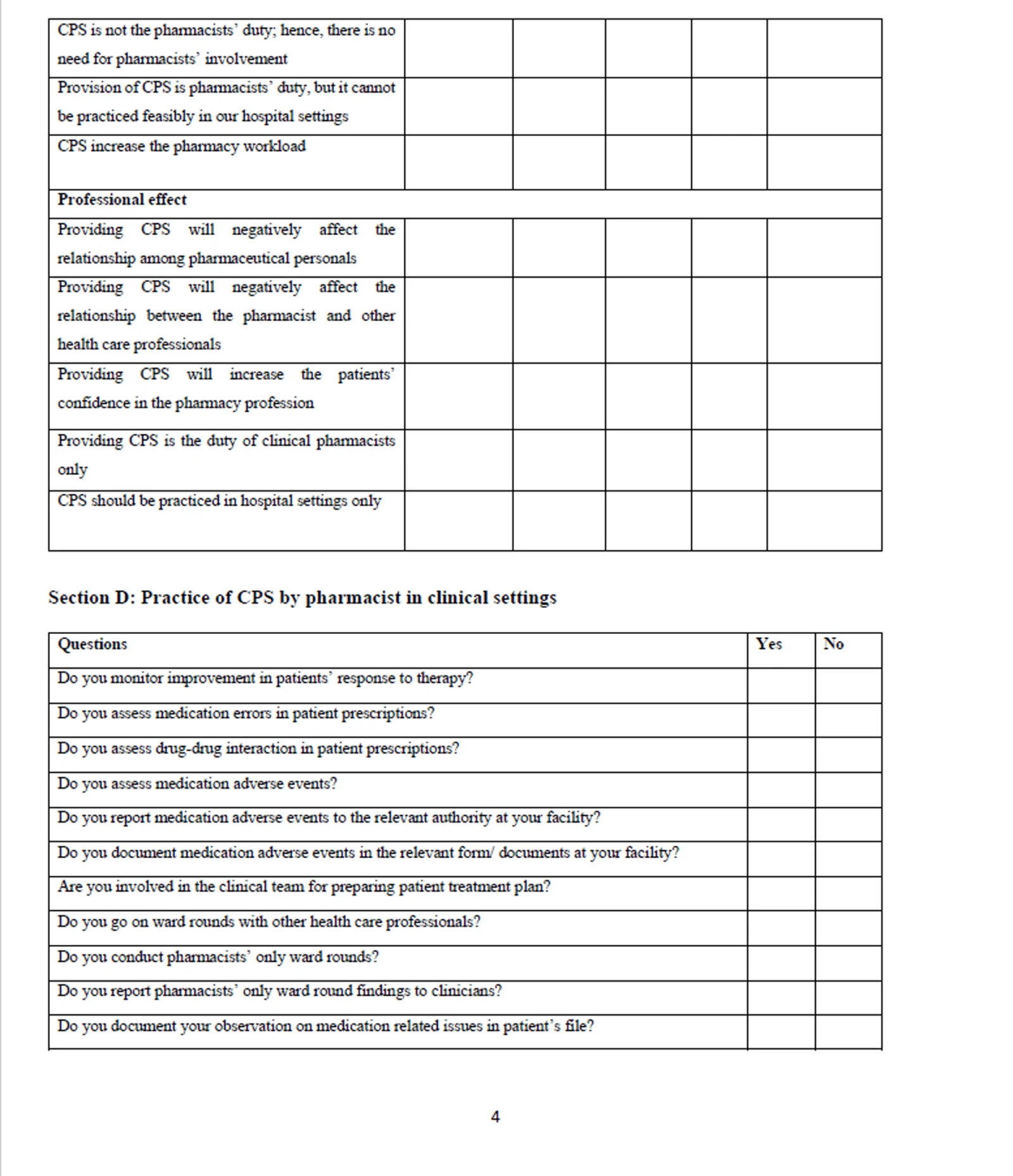
Questionnaire for pharmacists (page 4)

**Figure 8:**
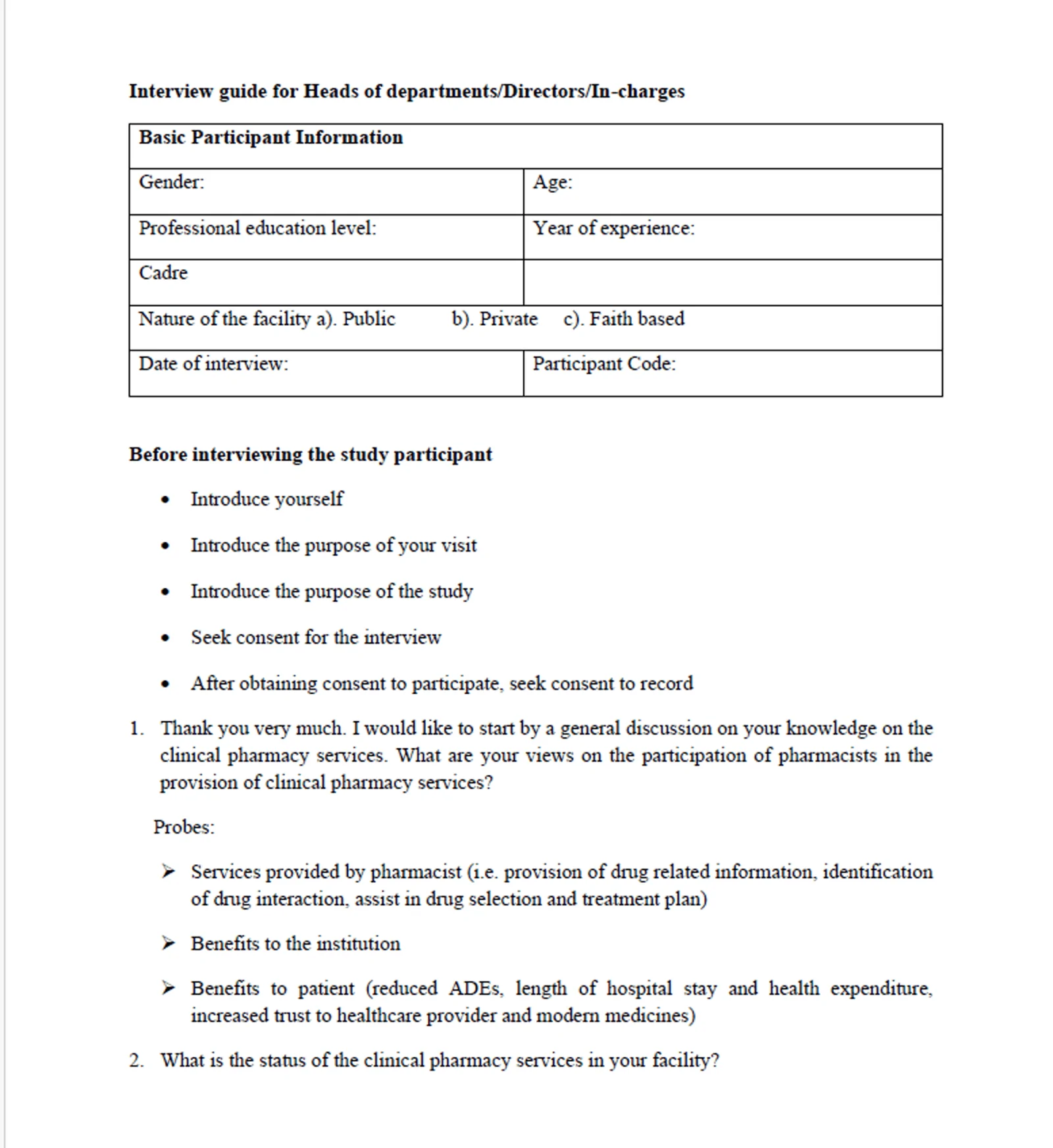
Semi-structured interview guide (page 1)

**Figure 9:**
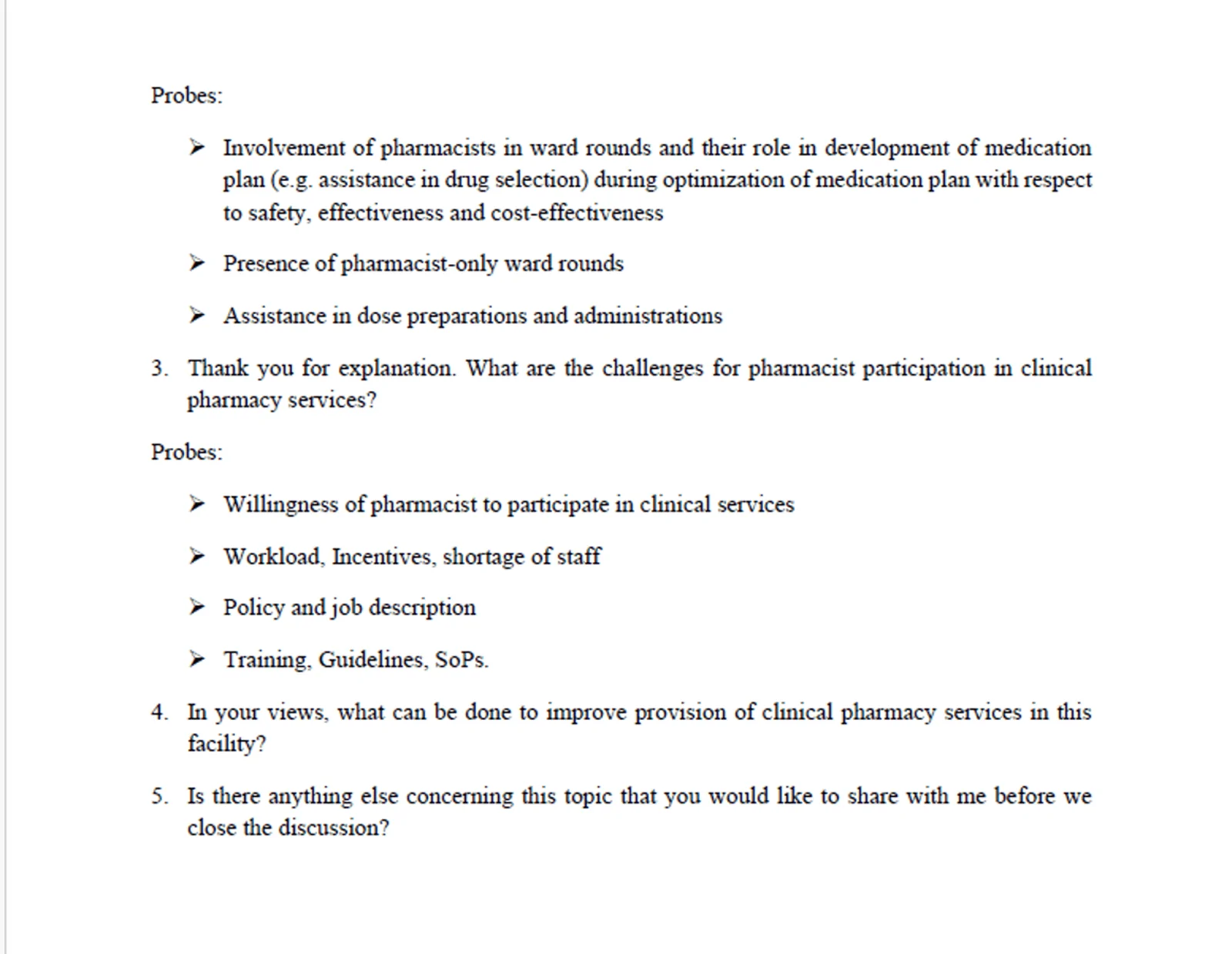
Semi-structured interview guide (page 2)

## Appendices

Appendix A: study questionnaire

Appendix B: semi-structured interview guide

Appendix C: focus group discussion guide

Before conducting the discussion, ➢ Introduce yourself ➢ Introduce the purpose of your visit ➢ Seek consent for the interview ➢ Ask participants to introduce themselves: o Age o Professional education level o Years of experience ➢ Record type and level of facility ➢ After obtaining consent to participate, seek consent to record.

1. Thank you very much for agreeing to participate in this short interview. I would like to start with a general discussion on your knowledge of the clinical pharmacy services. What do you understand about clinical pharmacy services? Probes: ➢ Patient-centered care (optimize medication use and promote health, wellness, and prevention of diseases) by .....: ➢ Activities incorporated in clinical pharmacy services (optimization of medication). ➢ Who qualifies to provide clinical pharmacy services (Pharmacist, Mpharm, PharmD, Others ....)? ➢ Importance of clinical pharmacy services

2. What is the status of the clinical pharmacy services in your facility? Probes: ➢ Participation of pharmacists in major ward rounds (schedule per week) ➢ Participation of pharmacists in Pharmacists-only service clinical rotations (schedule per week) ➢ Participation of Pharmacists in OPD clinics ➢ Dissemination of drug information in the clinical meetings ➢ Drug monitoring and evaluation on ADRs, therapeutic failure, dose individualization, and dose adjustment based on patient status) Thank you for the explanation.

3. What challenges/facilitators do you encounter when providing clinical pharmacy services? Probes: ➢ Willingness of the hospital administration to support clinical pharmacy services ➢ Resistance among pharmacists/pharmaceutical personnel ➢ Acceptability of clinical pharmacy services by other health care professionals, including medical doctors and nurses ➢ Increased workload ➢ Demotivation because of lack of officially recognized Incentives as other health workers, e.g. clinicians and nurses ➢ Shortage of staff ➢ Lack/availability of a job description that empowers a pharmacist to perform clinical pharmacy services at the hospital. ➢ Lack/availability of training to perform clinical Pharmacy services ➢ Lack/availability of guidelines and SOPs to guide the pharmacist to perform clinical Pharmacy services ➢ Incompetence of the pharmacists due to lack of clinical/therapeutic knowledge and skills, which hinders their participation in clinical pharmacy services ➢ Lack of specialized clinical pharmacists, example cardiac clinical pharmacist, nephrology clinical pharmacist, so many more

4. In your view, what can be done to improve provision of clinical pharmacy services in this facility?

5. Is there anything else concerning this topic that you would like to share with me before we close the discussion?
